# Effectiveness of advanced carbohydrate counting in type 1 diabetes mellitus: a systematic review and meta-analysis

**DOI:** 10.1038/srep37067

**Published:** 2016-11-14

**Authors:** Shimin Fu, Linjun Li, Shuhua Deng, Liping Zan, Zhiping Liu

**Affiliations:** 1 Department of Endocrinology, The First Affiliated Hospital of Chongqing Medical University, Chongqing, China; 2 Department of Cardiothoracic Intensive Care Unit, The First Affiliated Hospital of Chongqing Medical University, Chongqing, China

## Abstract

Potential benefits of carbohydrate counting for glycemic control in patients with type 1 diabetes mellitus (T1DM) remain inconclusive. Our aim is to systematically assess the efficacy of carbohydrate counting in patients with T1DM. We searched PubMed, Embase, Web of Science, Cochrane Library and the Chinese Biology Medicine (CBM) up to December 2015. Randomized controlled trials (RCTs) with at least 3 months follow-up that evaluated carbohydrate counting compared with usual or other diabetes dietary education in patients with T1DM were included. Overall meta-analysis identified a significant decrease in HbA_1c_ concentration with carbohydrate counting versus other diabetes diet method or usual diabetes dietary education (SMD: −0.35, 95%CI: −0.65 to −0.05, P = 0.023). Subgroup analysis restricted to trials which compared carbohydrate counting with usual diabetes dietary found a significant decrease in HbA_1c_ in carbohydrate counting group (SMD: −0.68, 95%CI: −0.98 to −0.38, P = 0.000), and a similar result has emerged from six studies in adults (SMD: −0.40, 95%CI: −0.78 to −0.02, P = 0.037). Carbohydrate counting may confer positive impact on glucose control. Larger clinical trials are warranted to validate this positive impact.

Type 1 diabetes mellitus (T1DM) is one of the most challenging medical disorders, and one of the key therapeutic goals to prevent or delay long-term diabetes complications in T1DM is the achievement and maintenance of near-normal glycemic control^1^. Only insulin treatment is not enough to rely on, dietary adjustments also play an important role in the regulation of blood glucose. Carbohydrates are a major determinant of postprandial blood glucose. Carbohydrate counting is a meal planning approach used with clients who have diabetes that focuses on carbohydrate as the primary nutrient affecting postprandial glycemic response. Advanced carbohydrate counting allows adjustment of the prandial insulin dose for actual carbohydrate intake in T1DM patients on intensive insulin therapy. Therefore, by calculating the carbohydrate amounts in each meal, insulin doses required to preserve postprandial blood glucose within normal limits can be predicted^2^,^3^. The current guidelines recommend that the algorithms for prandial insulin calculation take into account the carbohydrate amount of the meal^4^.

However, the efficacy of carbohydrate counting is not fully understood. At present, study to inquiry the effect of carbohydrate counting on T1DM patients is relatively little. Evidence from randomized controlled trials (RCTs) reported inconsistent results^5^,^6^,^7^. Previous systematic review^8^ or meta-analysis^9^ did not cite a clear conclusion. Furthermore, three recent trials^10^,^11^,^12^ with adequate power have been published and involve new evidence. Therefore, we performed this meta-analysis to evaluate the efficiency of carbohydrate counting on glycemic control in people with T1DM.

## Methods

### Literature search

This study was conducted following the Cochrane Collaboration and the PRISMA (Preferred Reporting Items for Systematic Reviews and Meta-Analyses statement) statement^13^,^14^. We identified relevant studies by searching the electronic databases in Pubmed, EMBASE, Cochrane Library, Web of Science and China Biology Medicine (CBM) for RCTs of carbohydrate counting from inception until December 2015. The search strategy included following terms: “carbohydrate counting”, “Type 1 diabetes mellitus”, “Glycated hemoglobin” and “HbA_lc_” (an example of specific strategy is shown in Supplementary material: Table S1). We read titles and abstracts of retrieved records to eliminate studies those were clearly irrelevant, and read full text of all remaining articles to decide eligible studies. Those discrepancies were resolved by discussion and consensus. Reference lists of identified trials and review articles were also hand-screened to identify any other relevant studies.

### Eligility criteria

Trials satisfying the following criteria were included: (1) design: randomised and quasi-randomised controlled clinical trials with at least 3 months’ follow-up; (2) population: T1DM who had injected insulin a minimum of three months; (3) intervention: carbohydrate counting versus other diabetes diet method or usual diabetes dietary education; (4) data: adequate information provided to calculate the standardized mean difference (SMD) and the corresponding 95% confidence interval (CI). We did not use any language limitations. Pregnant women with T1DM also be included in this study.

### Data extraction

Data was extracted and placed into a spreadsheet from each included study. The following information was collected: first author, year of publication, country of origin, number of patients, intervention, control, outcomes data (glycosylated hemoglobin (HbA_lc_) (%), hypoglycemia events, insulin dose and body mass index (BMI)) and follow up. We also contacted corresponding authors to verify extracted data and to request the missing data. The change in HbA_1c_ concentration was predefined primary outcome, and secondary outcomes were the change in hypoglycemia events, insulin dose and BMI.

### Risk of bias assessment

Risk of bias was assessed by using the Cochrane Collaboration’s tool^15^. Each study was assessed and scored as “high”, “low”, or “unclear” risk of bias to the following criteria: random sequence generation; allocation concealment; blinding of participants and personnel; blinding of outcome assessment; incomplete outcome data; selective reporting; and other bias. Blinding of patients and clinicians was extremely difficult and generally not feasible in these trials, and we judged that the primary outcome was less prone to be influenced by lack of blinding. Therefore, studies with high risk of bias for any one or more key domains except blinding were considered as at high risk of bias; while studies with low risk of bias for all key domains except blinding were considered as at low risk of bias; otherwise they were considered as at unclear risk of bias.

### Quality of evidence assessment

The quality of evidence for primary and secondary outcomes was assessed according to GRADE methodology for risk of bias, inconsistency, indirectness, imprecision, and publication bias; classified as high, moderate, low or very low. Summary tables were constructed by the GRADE system^16^,^17^,^18^,^19^ (GRADE version 3.6).

The literature search, data extraction, risk of bias assessment and evidence grade assessment were done independently by two authors (SF and LL) using a same approach. Disagreements were resolved by discussion among all authors.

### Statistical analysis

Since all the observation indexes are continuous, and the measurement time of outcome is inconsistent in different studies, thus we pooled the SMD with corresponding 95% CI by using the random-effects model. Heterogeneity across studies was explored by using the *I*^*2*^ statistic^20^ (the *I*^*2*^ > 50% indicated significant heterogeneity), and publication bias was assessed by using Begg’s test and Egger’s test (P < 0.05 was considered statistically significant for publication bias). Sensitivity analysis was conducted to investigate the stability and reliability of results.

## Result

### Trial selection and risk of bias assessment

**Figure 1:**
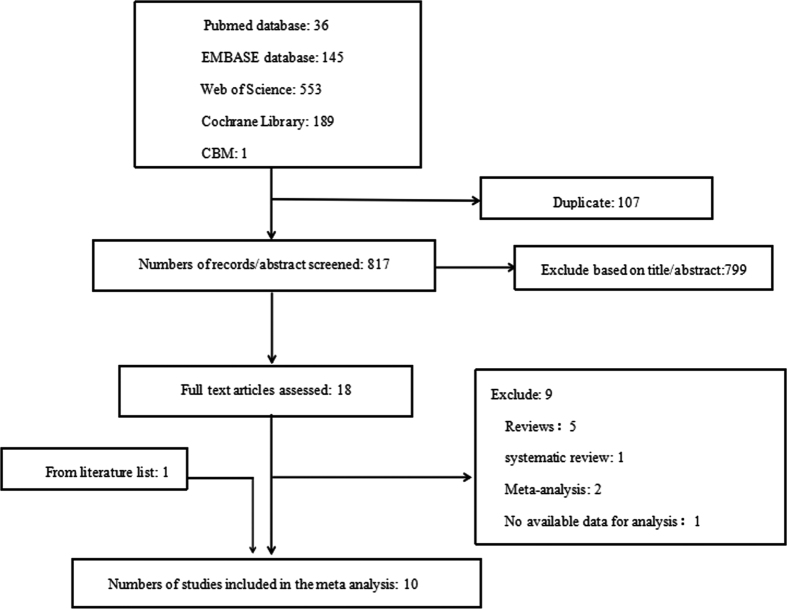
The flow diagram of literature review process.

**Figure 2:**
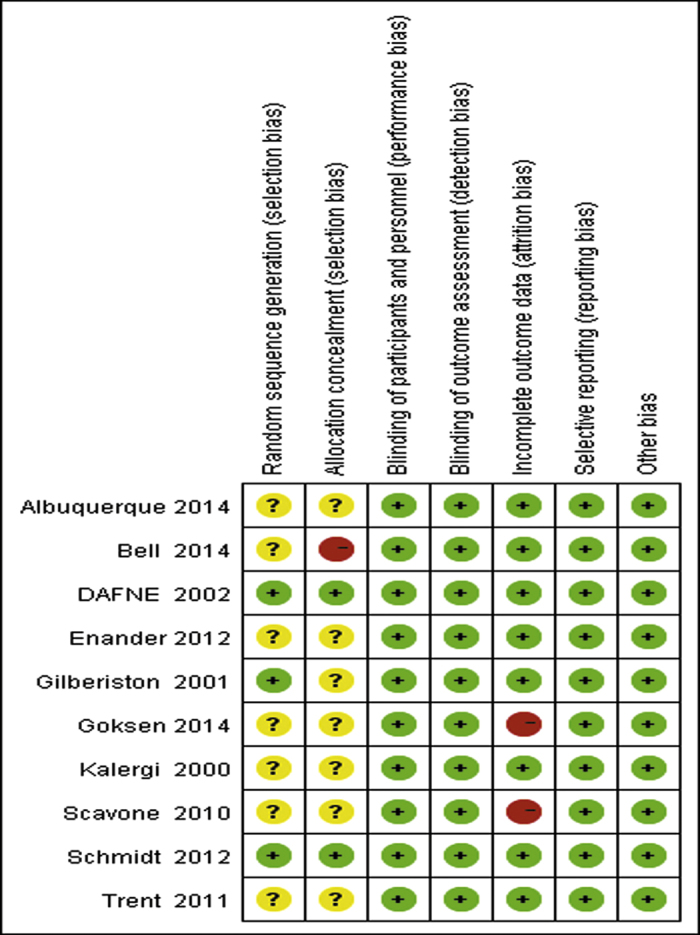
The result of risk of bias assessment: each risk of bias item for included studies (Green means low risk of bias, Yellow means unclear risk of bias, Red means high risk of bias).

**Figure 3:**
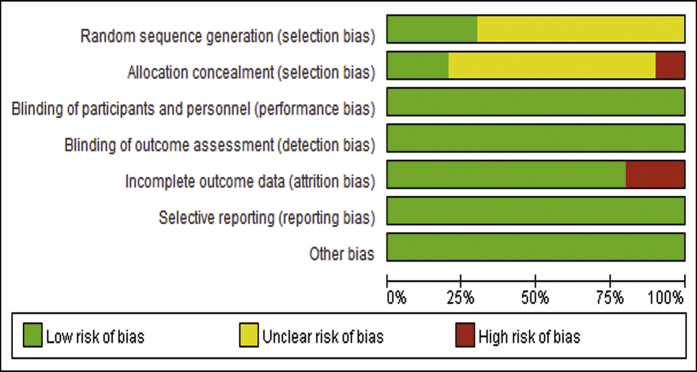
The result of risk of bias assessment: each risk of bias item showed as percentages across all included studies.

The initial search found 924 articles. After removing duplicates and screening the titles and abstracts, 18 articles were selected for full-text review, and 10 articles^5^,^6^,^7^,^10^,^11^,^12^,^21^,^22^,^23^,^24^ met the inclusion criteria. While one of them was excluded due to lack of essential data, and we failed to get the raw data from original author^25^. One more article^24^ from reference lists of identified trials also met the inclusion criteria and was included in this study. Totally 10 articles were included in the meta-analysis, the literature review process was showed in Fig. 1. According to the Cochrane Collaboration’s tool, two trials^9^,^24^ were categorized as at low risk of bias, five as at unclear^5^,^6^,^10^,^12^,^21^, and three as at high risk of bias^7^,^11^,^23^. All details of the risk of bias are supplied in Figs 2 and 3.

### Characteristics of articles

These ten studies involving 773 participants were published from 2000 to 2014. Four studies^5^,^7^,^10^,^12^ enrolled children and adolescents, and the remaining six studies included adults^6^,^11^,^21^,^22^,^23^,^24^. Among ten included studies, five compared the carbohydrate counting with other diabetes diet method^5^,^6^,^7^,^11^,^12^, and the remaining five compared the carbohydrate counting with usual diabetes dietary education^10^,^21^,^22^,^23^,^24^. All studies reported changes in HbA_1c_ concentration, four studies^5^,^7^,^12^,^21^ reported changes in daily insulin dosage, three studies^5^,^22^,^24^ reported changes in hypoglycemia event frequency and two studies^7^,^21^ reported changes in BMI. Detailed characteristics of eligible studies were provided in Table 1.

### Primary outcome

The primary outcome is HbA_1c_ concentration. All studies totaling 773 participants provided data on HbA_1c_ concentration. Compared with other diabetes diet method or usual diabetes dietary education, carbohydrate counting significantly reduced HbA_1c_ concentration (SMD: −0.35, 95%CI: −0.65 to −0.05, P = 0.023), with significant heterogeneity (*I*^*2*^ = 71.2%, *P* < 0.001). The heterogeneity among these studies could be related to different population and control group.

### Subgroup analysis and sensitivity analysis

**Figure 4:**
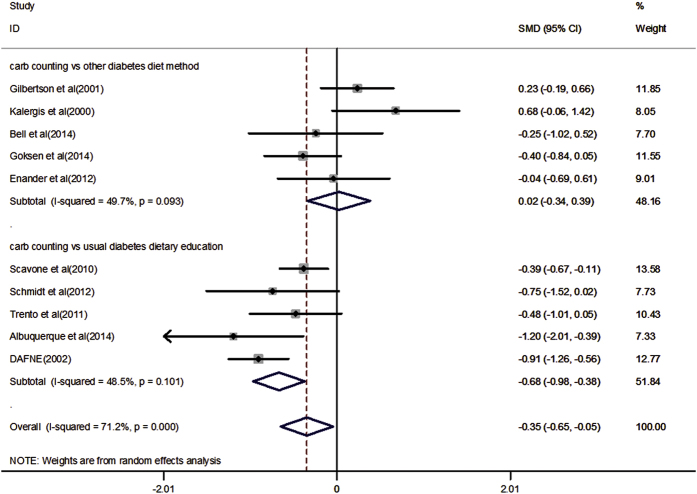
Subgroup analysis of HbA_1c_ concentration results according to different control group design.

**Figure 5:**
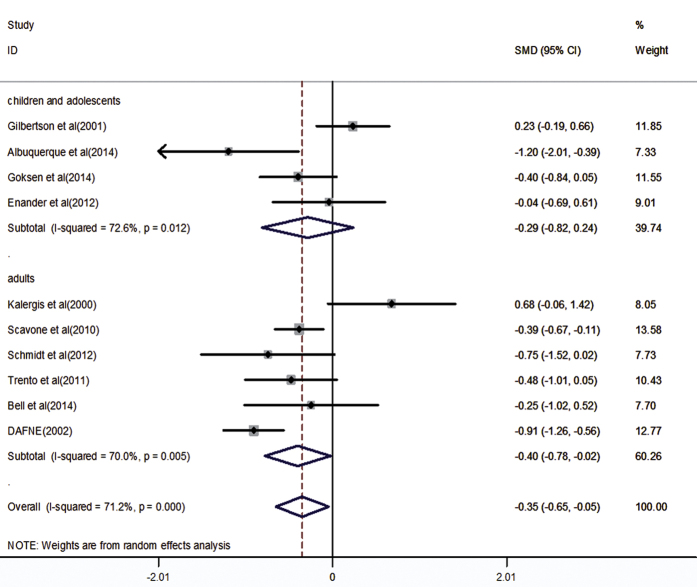
Subgroup analysis of HbA_1c_ concentration results according to different population.

We performed subgroup analysis according to population and control group. Results showed that compared with usual diabetes dietary education, carbohydrate counting significantly reduced HbA_1c_ concentration (SMD: −0.68, 95%CI: −0.98 to −0.38, P < 0.001), with no significant heterogeneity (*I*^*2*^ = 48.5%, *P* = 0.101). And a similar result has emerged from six studies in adults (SMD: −0.40, 95%CI: −0.78 to −0.02, P = 0.037), with significant heterogeneity (*I*^*2*^ = 70.0%, *P* = 0.005). All results of subgroup analyses are presented in Figs 4 and 5. And sensitivity analysis showed that present results possess superior reliability (Supplementary material: Figure S1).

### Secondary outcomes

**Figure 6:**
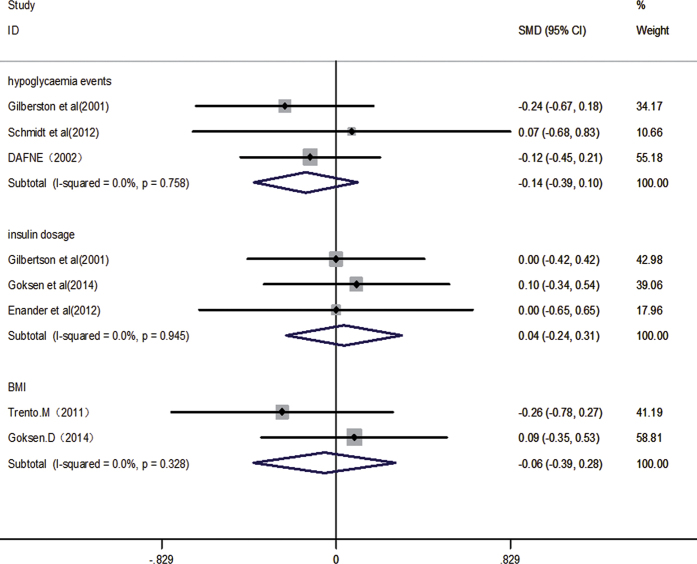
Effect of carbohydrate counting versus other diabetes diet method or usual diabetes dietary education for reducing hypoglycaemia events, insulin dosage and BMI.

Secondary outcomes including the change in hypoglycemia events, insulin doses and BMI. There are four studies^5^,^7^,^12^,^21^ reported insulin doses, while the data one study reported was suspectable^21^, and we failed to obtain raw data from authors. Thus it was excluded and three studies were included in the meta-analysis. There are three studies^5^,^22^,^24^ reported hypoglycemia events and three reported BMI data^7^,^21^, respectively. Compared with other diabetes diet method or usual diabetes dietary education, carbohydrate counting did not significantly reduce the hypoglycaemia events (SMD: −0.14, 95%CI: −0.39 to 0.10, P = 0.254; *I*^*2*^ = 0.0%, *P* = 0.758), insulin dosage (SMD: 0.04, 95%CI: −0.24 to 0.31, P = 0.788; *I*^*2*^ = 0.0%, *P* = 0.945) or BMI (SMD: −0.06, 95%CI: −0.39 to 0.28, P = 0.749; *I*^*2*^ = 0.0%, *P* = 0.328) (Fig. 6).

### Strength of evidence and publication bias

The quality of evidences was evaluated by GRADE system. The level of evidence was at level B and moderate recommendation for HbA_1c_ concentration. All evidence profiles for the primary and secondary outcomes were provided in Table 2. For the meta-analysis of carbohydrate counting on HbA_1c_ concentration, no publication bias was observed by Begg’s test and Egger’s test (Begg’s, P = 0.721; Egger’s, P = 0.688) (Supplementary material: Figure S2).

## Discussion

### Main findings

This meta-analysis systematically reviewed the current available literature and found that (1) In general, compared with other diabetes diet method or usual diabetes dietary, carbohydrate counting significantly reduced HbA_1c_ concentration, evidence of this benefit was consistent in previous meta-analysis. While subgroup analysis restricted to trials which compared carbohydrate counting with other diabetes diet method found no significant decrease in HbA_1c_ concentration in carbohydrate counting group. Comparing carbohydrate counting with other dietary method is in fact examining the impact of carbohydrate counting plus education in a more general sense, thus the efficiency of carbohydrate counting on glycemic control might be exaggerated. (2) We found that carbohydrate counting significantly reduced HbA_1c_ concentration in adult group, while not in children and teenagers group. It may be because that adults are more likely to learn and apply carbohydrate counting.

### Comparison with previous meta-analysis

In our study, the effect of carbohydrate counting reducing HbA_1c_ concentration is consistent with previous meta-analysis^11^. While differences between our study and previous analysis should be noted. First, previous meta-analysis included seven trials totaling 703 participants. We included six of the seven trials, the other one was excluded due to lack of essential data, and we failed to get the raw data from authors. While we added four new trials, and we also added subgroup analysis according to the control group, got a more stable and reliable conclusion by eliminating interference factors. Our meta-analysis found that heterogeneity among trials mainly is from the design of different control group, rather than population. In addition, we evaluated the quality of evidence and the strength of recommendations. Therefore, our current meta-analysis was the latest and the most comprehensive one.

### Guidance for clinical practice

First, our study found that carbohydrate counting has a positive effect on reducing HbA_1c_ concentration. This effect is stable and reliable, and carbohydrate counting should be recommended for the routine treatment of T1DM. Second, up to now, little attention was paid to the study of carbohydrate counting’s effect on hypoglycemia events, insulin doses and BMI. The impact of carbohydrate counting on these aspects is a direction of future research. Finally, considering the dietary education in a more general sense may exaggerate the effect of carbohydrate counting, more clinical trials compared carbohydrate counting with dietary education in a more general sense are warranted to validate the positive impact of carbohydrate counting.

### Limitations

Our study also has limitations. Though high quality of studies included in this meta-analysis, the sample sizes of these studies are small, and there is significant heterogeneity among studies, the reliability of results can be affected. More high quality trials with large samples are needed to confirm current results.

## Conclusion

Our meta-analysis suggested that carbohydrate counting plays an important role in reducing HbA_1c_ concentration, and this positive impact still needs evaluation by high-quality randomly controlled experiments.

## Additional Information

**How to cite this article**: Fu, S. *et al*. Effectiveness of advanced carbohydrate counting in type 1 diabetes mellitus: a systematic review and meta-analysis. *Sci. Rep.* **6**, 37067; doi: 10.1038/srep37067 (2016).

**Publisher's note:** Springer Nature remains neutral with regard to jurisdictional claims in published maps and institutional affiliations.

## Supplementary Material

Supplementary Information

## Figures and Tables

**Table 1:** Characteristics of studies included in the systematic review and meta-analysis.

Author/year	Country	Population	No. of patients	Intervention	Control	HbA_lc_ (%) (M ± SD) intervention/Control	Hypoglycemia (M ± SD)	Insulin dose (U/kg) (M ± SD)	BMI (M ± SD)	Follow up
Gilbertson *et al*.^5^	Australia	Children	104; 38/49;51/55	15 g CHO exchanges for each meal and snack	Low glycemic index diet	8.60 ± 1.40 to 8.60 ± 1.40 8.30 ± 1.30 to 8.00 ± 1.00	7.30 ± 5.70 to 5.80 ± 5.50 6.90 ± 6.20 to 6.90 ± 6.80	0.90 ± 0.30 to 1.00 ± 0.30 1.00 ± 0.30 to 1.10 ± 0.30	—	12 months
Kalergis *et al*.^6^	Canada	Adults	21; 15/21;15/21	carbohydrate counting with qualitative adjustment of insulin for exercise and stress (1Uinte/10 g ratio)	food exchanges with qualitative adjustment of insulin for exercise and stress	0.14 ± 0.63/−0.82 ± 0.63 (mean change ± standard error)	—	—	—	3.5 months
Scavone *et al*.^24^	Rome	Adults	256; 73/100;156/156	Carbohydrates counting education (4-week), reassessed every 3 months	Usual care	7.80 ± 1.30 to 7.40 ± 0.90 7.50 ± 0.80 to 7.50 ± 1.10	—	—	—	9 months
Schmidt *et al*.^23^	Denmark	Adults	63; 43/54;8/9	group diabetes education and carbohydrate counting education (1-h session, two 15-min telephone consultations,individual 1-h follow-up consultation)	group diabetes education (food recommendations, self-monitoring techniques, estimate insulin doses)	9.00 ± 0.68 to 8.25 ± 0.70 9.10 ± 0.70 to 8.90 ± 1.10	2.40 ± 1.20 to 1.89 ± 1.18 2.40 ± 1.30 to 1.80 ± 1.40	—	—	16 weeks
Trento *et al*.^22^	Italy	Adults	56; 27/27; 29/29	Carbohydrate counting programme (8-session) and usual group care	Usual diabetes education and group care	7.60 ± 1.30 to 7.20 ± 0.90 7.70 ± 1.24 to 7.90 ± 1.40	—	—	24.4 ± 2.6 to 23.4 ± 5.3 23.5 ± 3.3 to 23.5 ± 2.9	30 months
Bell *et al*.^11^	Australia	Adults	26; 13/13;13/13	Group education and individual sessions (carbohydrate counting)	Group education and individual sessions (Food Insulin Index)	8.60 ± 0.90 to 8.30 ± 0.60 8.10 ± 0.70 to 8.00 ± 0.90	—	—	—	12 weeks
Albuquerque *et al*.^10^	Brasil	adolescents	28; 14/14;14/14	Nutritional counseling (carbohydrate counting)	Usual nutritional counseling	10.59 ± 3.43 to 8.39 ± 2.28 8.42 ± 2.14 to 9.62 ± 2.91	—	—	—	4 months
Goksen *et al*.^7^	Turkey	children and adolescents	110; 52/55;32/55	carbcounting group education (2-week)	traditional exchange-based meal plan	8.10 ± 1.00 to 7.87 ± 1.38 8.43 ± 1.52 to 8.76 ± 1.77	—	0.92 ± 0.29 to 1.01 ± 0.28 0.96 ± 0.36 to 1.02 ± 0.31	19.61 ± 3.22 to 20.81 ± 3.38 20.89 ± 3.31 to 21.80 ± 3.68	2 years
DAFNE^24^	England	Adults	169; 68/84;72/85	carbohydrate group education (5-day, adjust insulin to suit lifestyle)	Usual care	9.40 ± 1.20 to 8.40 ± 1.20 9.30 ± 1.10 to 9.40 ± 1.30	2.04 ± 1.20 to 2.16 ± 1.3 2.12 ± 1.40 to 2.40 ± 1.3	—	—	6 months
Enander *et al*.^12^	Sweden	children and young people	45; 26/30; 14/15	dietary education in carbohydrate counting	dietary education in the traditional methodology (the plate exchange method)	7.43 ± 0.83 to 7.69 ± 1.00 7.70 ± 1.00 to 8.00 ± 1.00	—	0.78 ± 0.24 to 0.80 ± 0.19 0.81 ± 0.22 to 0.83 ± 0.22	—	12 months

HbA_lc_: glycosylated Hemoglobin; M:mean; SD: standard deviation; BMI: body mass index; CHO: carbohydrates.

**Table 2:** GRADE evidence profile for the effectiveness of advanced carbohydrate counting in type 1 diabetes mellitus.

Quality assessment							No of patients		Effect		Quality	Importance
No of studies	Design	Risk of bias	Inconsistency	Indirectness	Imprecision	Other considerations	Carbohydrate counting	Other diabetes diet method or usual diabetes dietary education	Relative (95% CI)	Absolute		
**HbA**_**lc**_ **(follow-up 3 to 30 months; measured with: Blood test; range of scores: 7–9; Better indicated by lower values)**												
10	randomised trials	serious	serious	no serious indirectness	no serious imprecision	strong association	369	404	—	SMD 0.35 lower (0.65 lower to 0.05 lower)	⊕⊕⊕Ο MODERATE	CRITICAL
**hypoglycaemic (follow-up 4 to 12 months; measured with: recall; range of scores: 4–6; Better indicated by lower values)**												
3	randomised trials	no serious risk of bias	no serious inconsistency	no serious indirectness	serious	reporting bias strong association	149	131	—	SMD 0.14 lower (0.39 lower to 0.1 higher)	⊕⊕⊕Ο MODERATE	IMPORTANT
**insulin dose (follow-up 12 to 24 months; measured with: record and calculation; range of scores: 4–6; Better indicated by lower values)**												
3	randomised trials	serious	no serious inconsistency	no serious indirectness	serious	reporting bias strong association	116	97	—	SMD 0.04 higher (0.24 lower to 0.31 higher)	⊕⊕ΟΟ LOW	IMPORTANT
**BMI (follow-up mean 24 to 30 months; measured with: calculation; range of scores: 4–6; Better indicated by lower values)**												
2	randomised trials	serious	no serious inconsistency	no serious indirectness	no serious imprecision	reporting bias strong association	79	61	—	SMD 0.06 lower (0.39 lower to 0.28 higher)	⊕⊕⊕Ο MODERATE	IMPORTANT
