# Supplementary material for: Effectiveness of advanced carbohydrate counting in type 1 diabetes mellitus: a systematic review and meta-analysis
Source: Sci Rep. 2016 Nov 14;6:37067. doi: 10.1038/srep37067 (PMC5107938; doi:10.1038/srep37067)
Supplement: Supplementary Information [file srep37067-s1.pdf]

Effectiveness of advanced carbohydrate counting in type 1 diabetes mellitus : a systematic review and meta-analysis

Shimin Fu[1,†], Linjun Li[2,†], Shuhua Deng[1], Liping Zan[1] and Zhiping Liu[1*]

[1]Department of Endocrinology, The First Affiliated Hospital of Chongqing Medical University, Chongqing, China

[2]Department of Cardiothoracic Intensive Care Unit, The First Affiliated Hospital of Chongqing Medical University, Chongqing, China

[†] These authors contributed equally to the study.

[*] Corresponding author: Zhiping Liu, fax +86 023 89011552, email nfmlzp@163.com.

PRISMA 2009 Flow Diagram

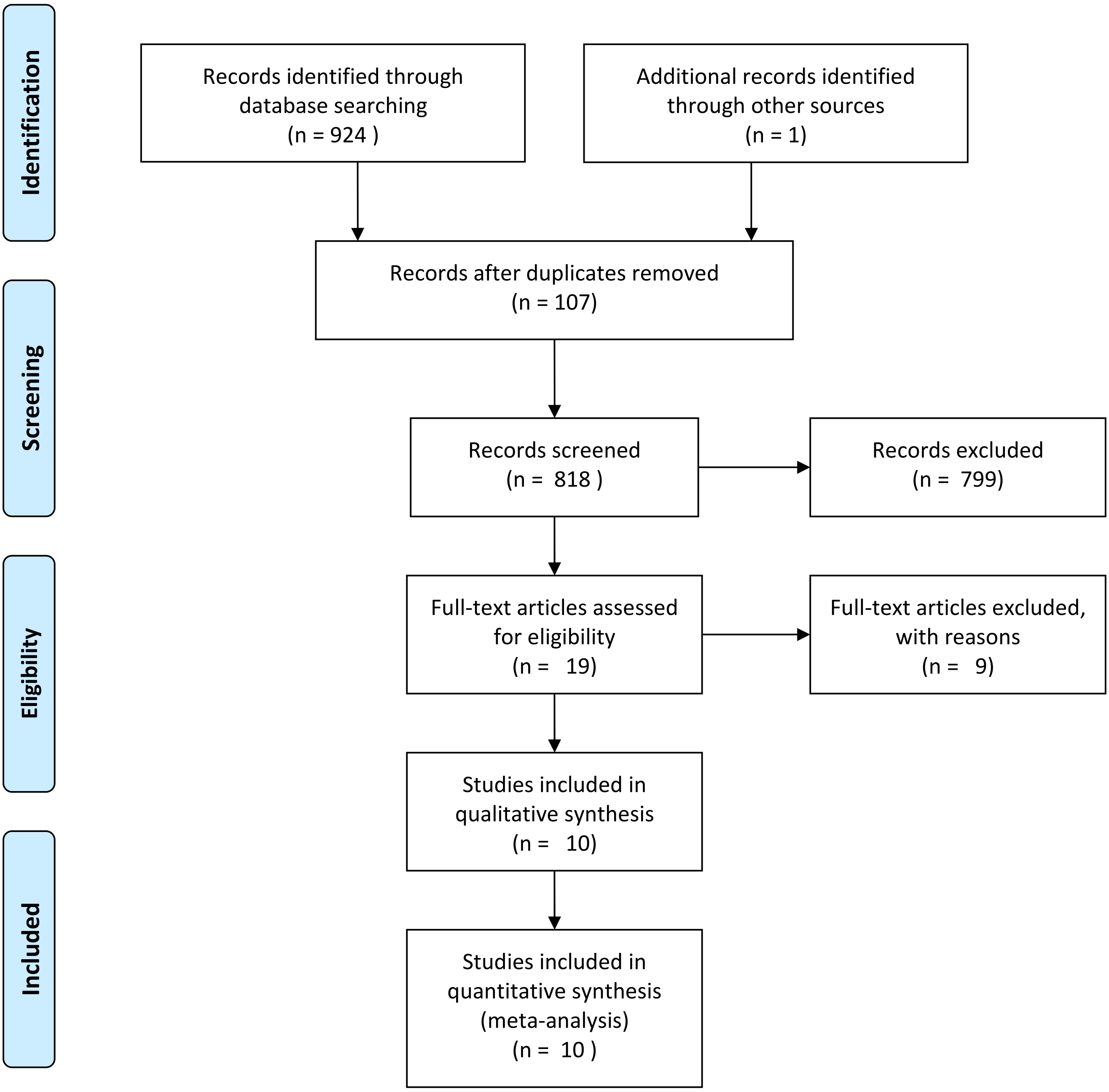

From: Moher D, Liberati A, Tetzlaff J, Altman DG, The PRISMA Group (2009). *P*referred *R*eporting *I*tems for *S*ystematic Reviews and *M*eta-*A*nalyses: The PRISMA Statement. PLoS Med 6(6): e1000097. doi:10.1371/journal.pmed1000097

For more information, visit www.prisma-statement.org.

Figure S1.Sensitivity analyses SMD of HbA_{1c} concentration in all eligible studies.

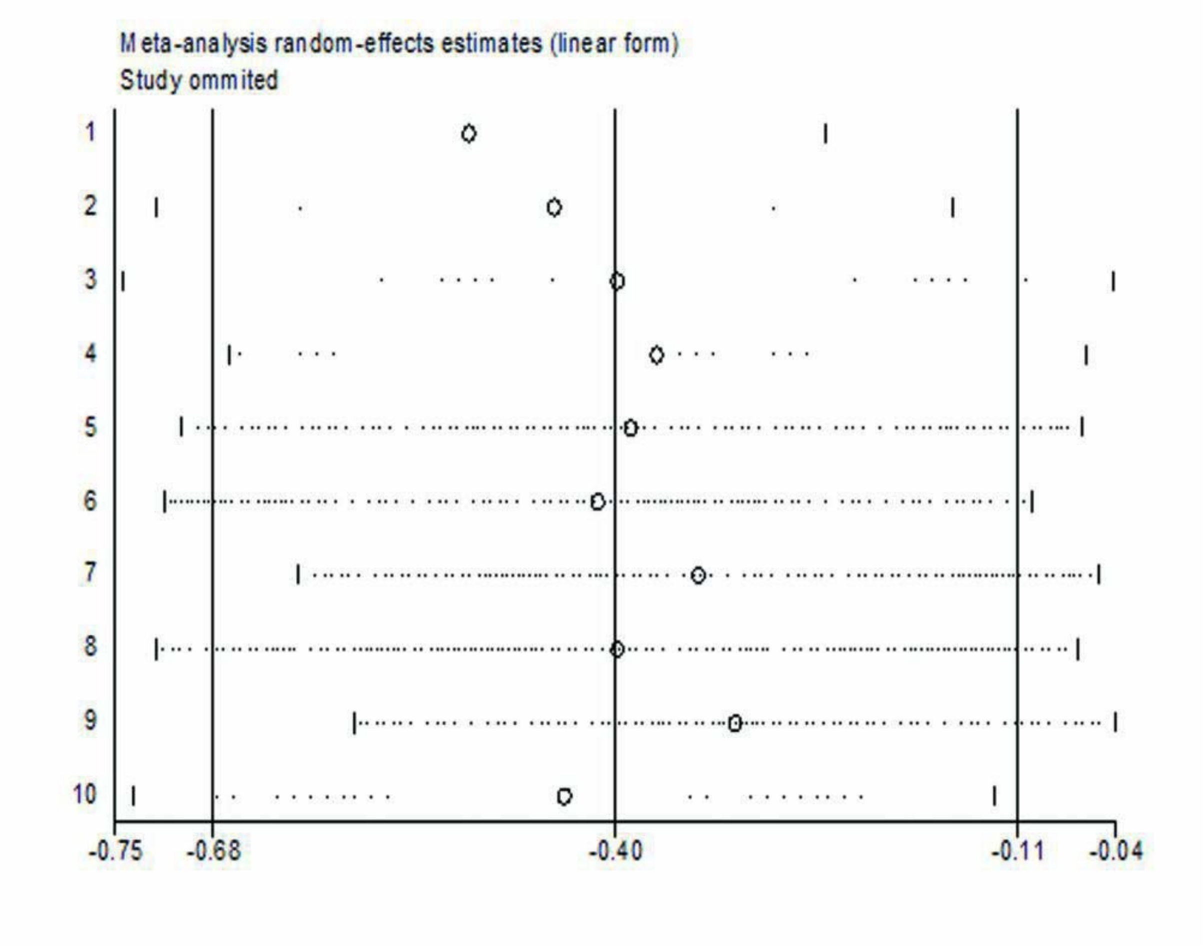

Figure S2. Egger's test for assessing publication bias for HbA_{1c} concentration.

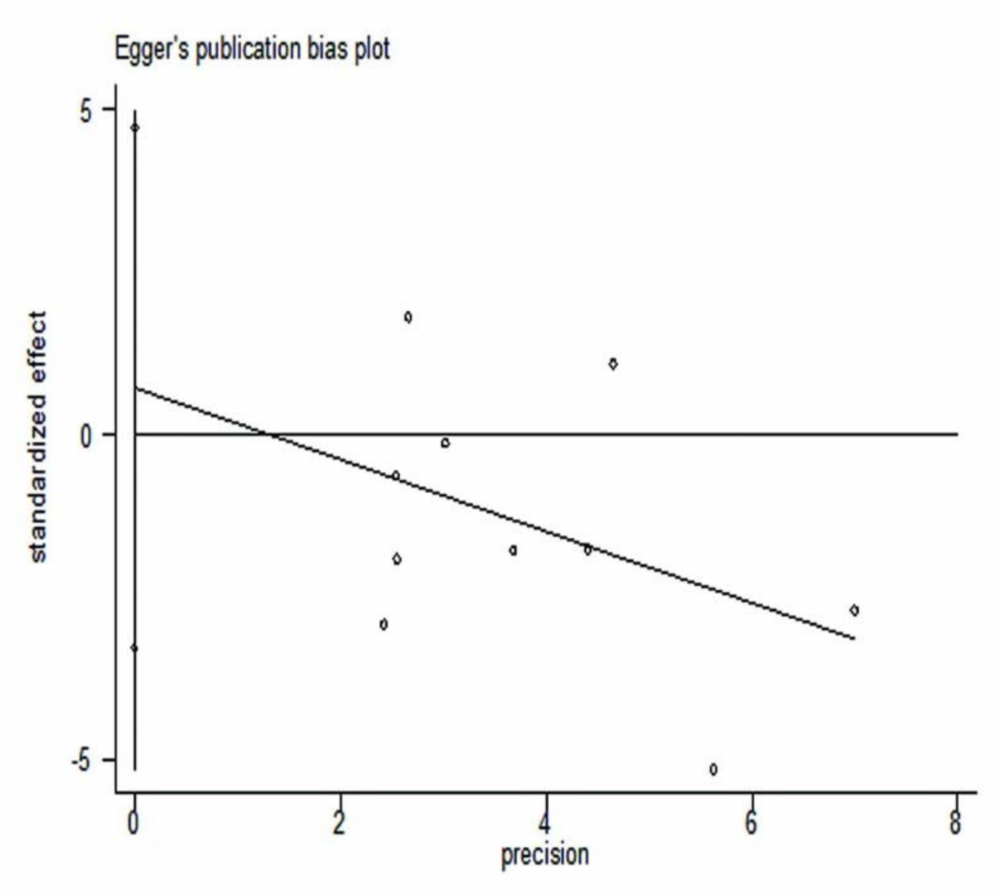

Table S1-The retrieval strategy In the pubmeddatabase.

Search	Query	Items found
#10	Search (((("Hemoglobin A, Glycosylated"[Mesh]) OR ((HbAIC[Title/Abstract]) OR ((((((Glycated hemoglobin[Title/Abstract]) OR glycosylated hemoglobin[Title/Abstract]) OR hemoglobin A1C[Title/Abstract]) OR saccharified hemoglobin[Title/Abstract]) OR HA1C[Title/Abstract])))) AND ((("Diabetes Mellitus, Type 1"[Mesh]) OR ((((Type 1 diabetes[Title/Abstract]) OR Type 1 diabetes mellitus[Title/Abstract]) OR insulin dependent diabetes mellitus[Title/Abstract]) OR T1DM[Title/Abstract])))) AND ((((((carb counting[Title/Abstract]) OR carb-counting[Title/Abstract]) OR carbohydrate counting[Title/Abstract]) OR dietary carbohydrates[Title/Abstract]) OR Carbohydrate exchange[Title/Abstract]))	36
#9	Search ("Hemoglobin A, Glycosylated"[Mesh]) OR ((HbAIC[Title/Abstract]) OR ((((((Glycated hemoglobin[Title/Abstract]) OR glycosylated hemoglobin[Title/Abstract]) OR hemoglobin A1C[Title/Abstract]) OR saccharified hemoglobin[Title/Abstract]) OR HA1C[Title/Abstract]))	32487
#8	Search "Hemoglobin A, Glycosylated"[Mesh]	24554
#7	Search (HbAIC[Title/Abstract]) OR ((((((Glycated hemoglobin[Title/Abstract]) OR glycosylated hemoglobin[Title/Abstract]) OR hemoglobin A1C[Title/Abstract]) OR saccharified hemoglobin[Title/Abstract]) OR HA1C[Title/Abstract])	14779
#6	Search HbAIC[Title/Abstract]	67
#5	Search (((((Glycated hemoglobin[Title/Abstract]) OR glycosylated hemoglobin[Title/Abstract]) OR hemoglobin A1C[Title/Abstract]) OR saccharified hemoglobin[Title/Abstract]) OR HA1C[Title/Abstract]	14726

#4	Search (("Diabetes Mellitus, Type 1"[Mesh]) OR ((((Type 1 diabetes[Title/Abstract]) OR Type 1 diabetes mellitus[Title/Abstract]) OR insulin dependent diabetes mellitus[Title/Abstract]) OR T1DM[Title/Abstract]))	79035
#3	Search "Diabetes Mellitus, Type 1"[Mesh]	63341
#2	Search ((((Type 1 diabetes[Title/Abstract]) OR Type 1 diabetes mellitus[Title/Abstract]) OR insulin dependent diabetes mellitus[Title/Abstract]) OR T1DM[Title/Abstract])	41995
#1	Search (((((carb counting[Title/Abstract]) OR carb-counting[Title/Abstract]) OR carbohydrate counting[Title/Abstract]) OR dietary carbohydrates[Title/Abstract]) OR Carbohydrate exchange[Title/Abstract])	1250
